# DRESS Syndrome Secondary to Carbamazepine Therapy Presenting with Bilateral Acute Anterior Uveitis and Angle Closure Glaucoma

**DOI:** 10.18502/jovr.v14i3.4795

**Published:** 2019-07-18

**Authors:** Divya Karuppannasamy, Raghuram Andavar, Jayavardhana Arumugam, Kumaresan Muthuvel

**Affiliations:** ^1^Department of Ophthalmology, PSG Institute of Medical Sciences and Research, Coimbatore, Tamil Nadu, India; ^2^Department of Paediatrics, PSG Institute of Medical Sciences and Research, Coimbatore, Tamil Nadu, India; ^3^Department of Dermatology, PSG Institute of Medical Sciences and Research, Coimbatore, Tamil Nadu, India

**Keywords:** Anterior Uveitis, Carbamazepine, DRESS Syndrome

## Abstract

**Purpose:**

Drug reaction with eosinophilia and systemic symptoms (DRESS) syndrome is a rare, life-threatening multi-system adverse drug reaction characterized by febrile skin rash, hematologic abnormalities, and involvement of internal organs. We report a case of DRESS syndrome in a child presenting with primary ophthalmic manifestations.

**Case Report:**

An 11-year-old boy presented with severe pain and diminished vision in both eyes six weeks after starting carbamazepine therapy for seizure disorder. Ocular examination revealed features of bilateral acute anterior uveitis, acute onset myopia, and angle closure glaucoma secondary to uveal effusion. Additionally, the patient was febrile with a generalized maculopapular rash, and blood investigations revealed eosinophilic leukocytosis. A diagnosis of carbamazepine-induced DRESS syndrome was made, and carbamazepine therapy was discontinued. Treatment with cycloplegics, topical, and systemic steroids resulted in prompt clinical recovery.

**Conclusion:**

Ophthalmologists should be aware that hypersensitivity to anticonvulsants, such as carbamazepine, can present with bilateral uveitis and uveal effusion along with systemic symptoms. Prompt diagnosis and treatment can prevent vision loss and life-threatening complications. Patients should be counselled about potential adverse effects of anticonvulsants before therapy.

##  INTRODUCTION

Drug reaction with eosinophilia and systemic symptoms (DRESS) syndrome is a rare, potentially fatal idiosyncratic reaction characterized by cutaneous manifestations, hematological changes, and involvement of internal organs.^[1]^ The incidence of this syndrome ranges from 1 in 1000 to 1 in 10,000 drug exposures. The liver is the most commonly affected visceral organ.^[2]^ Primary ocular involvement is uncommon, and only a few cases have been reported in adults.^[3,4,5,6,7]^ Here, we report the occurrence of bilateral acute anterior uveitis and angle closure glaucoma associated with carbamazepine-induced DRESS syndrome in a child.

##  CASE REPORT

An 11-year-old boy was admitted at the ophthalmology department with complaints of severe pain and diminished vision in both eyes for two days. He had never worn glasses, and his ocular history was unremarkable. Six weeks earlier, he had been diagnosed with a seizure disorder by a pediatrician; oral carbamazepine therapy (200 mg twice daily) was started. Three weeks before the onset of ocular symptoms, he had developed fever and generalized skin rash, which was diagnosed as a viral exanthem and managed with supportive therapy.

Ophthalmic examination revealed a best corrected visual acuity of 20/120 in the right eye and 20/60 in the left eye with -9.0 diopters spherical (DSph) and -1.0 diopters cylinder (DCyl) at 90${}^{\text{o}}$ in the right eye and -10.0 DSph and -1.0 DCyl at 90${}^{\text{o}}$ in the left eye. The eyelids were swollen, and chemosis was noted in both eyes. Anterior segment examination revealed corneal epithelial edema, shallow anterior chamber with 3 plus cells, sluggishly reacting pupil, and clear crystalline lens in both eyes. Intraocular pressure by Goldmann applanation tonometry was 45 and 40 mmHg in the right and left eyes, respectively. Gonioscopy revealed bilateral 360${}^{\text{o}}$ appositional angle closure, and no angle structures were visible. Fundus examination revealed normal optic disc and vessels with a cup-to-disc ratio of 0.3. The axial length of the right eye was 21.91 mm and that of the left eye was 22.05 mm. Considering the acute onset of myopia, bilateral shallow anterior chambers, and angle-closure glaucoma, ciliochoroidal effusion, probably due to carbamazepine, was diagnosed.

**Figure 1:**
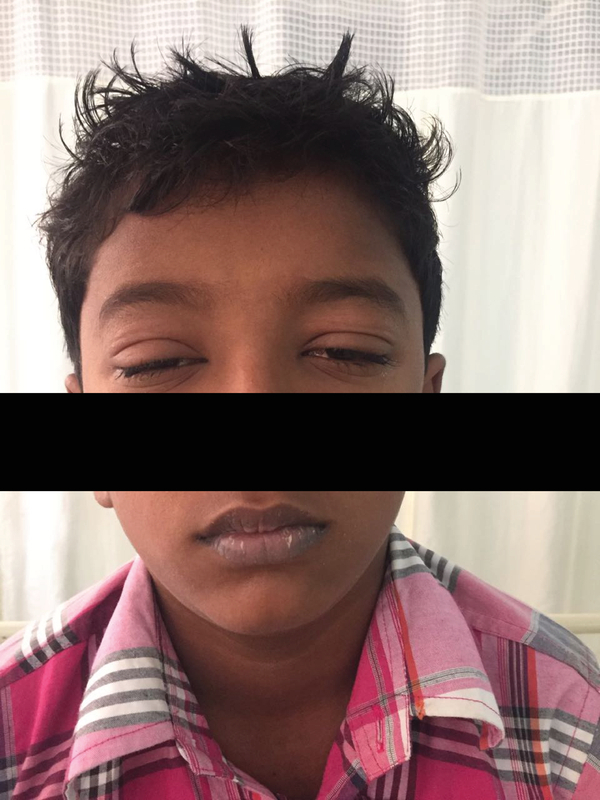
Clinical photograph showing swollen eyelids and crusted lesions on lips.

In addition to the ocular findings, the child was febrile with a temperature of 39.8${}^{\text{o}}$C and had a generalized maculopapular rash. The lips were swollen with crusted lesions [Figure 1]. Systemic examination revealed no abnormalities. Investigations revealed leukocytosis ($18.9\times 10^{3}$/μL) with increased neutrophils ($14.0\times 10^{3}$/μL) and eosinophils ($1.8\times 10^{3}$/μL). Peripheral smear showed a few atypical lymphocytes and no blast cells. Hemoglobin level was 12.9 g/dL, and platelet count was $358\times 10^{3}$/μL. Blood culture tests were negative, and liver function tests were within normal limits. A dermatologist was consulted for the skin lesions, and DRESS syndrome was diagnosed.

**Figure 2:**
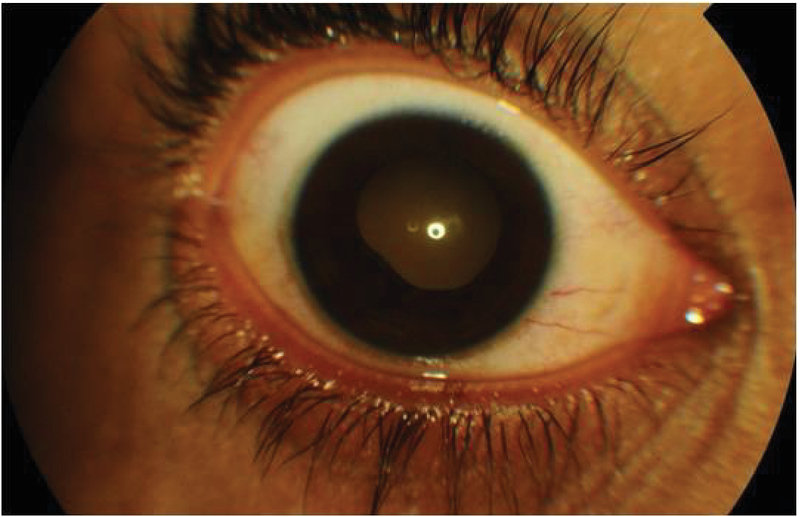
Anterior segment photograph of the right eye showing posterior synechiae.

**Figure 3:**
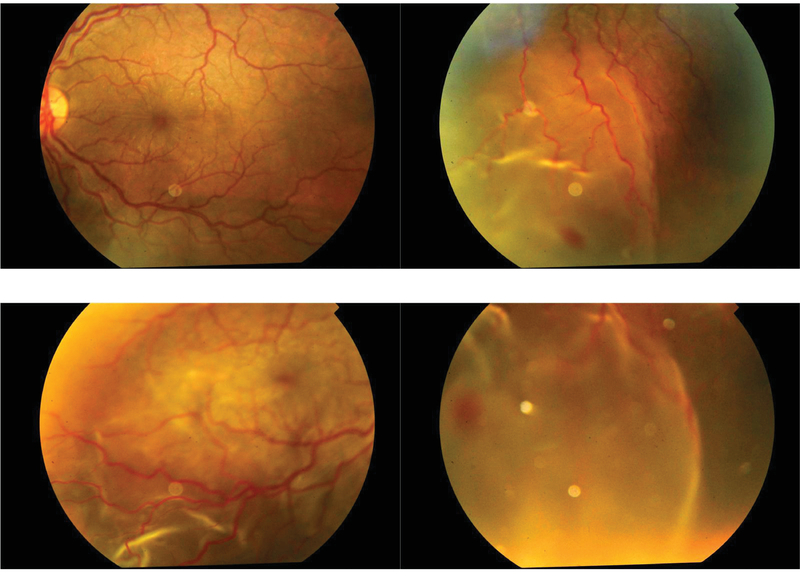
Fundus photograph of both eyes showing choroidal folds at the macula, retinal hemorrhages, and serous retinal detachment.

Carbamazepine was discontinued and substituted with levetiracetam. Treatment was initiated with homatropine, timolol, and topical steroids, along with intravenous mannitol (1 g/kg body weight). His symptoms improved dramatically after two doses of intravenous dexamethasone (4 mg). Ocular examination on the third day revealed a clear cornea, deep anterior chamber with over three cells in both eyes, and posterior synechiae in the right eye [Figure 2]. Intraocular pressure in the right and left eyes was 6 and 5 mmHg, respectively. Fundus examination revealed choroidal folds in the macula and serous elevation of the retina along with scattered retinal hemorrhages [Figure 3].

**Figure 4:**
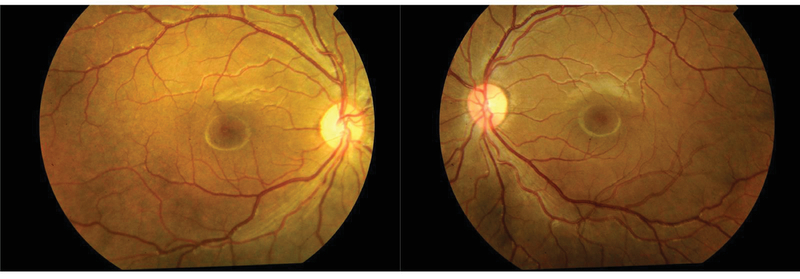
Fundus photograph at the three-week follow-up showing normal optic disc and macula.

Along with topical steroids and homatropine, oral prednisolone therapy was started (initial dose, 1 mg/kg body weight) and subsequently tapered. At the three-week follow-up, uveitis and retinal hemorrhages had resolved [Figure 4]. Unaided visual acuity was 20/20, and intraocular pressure was 16 mm Hg in both eyes.

##  DISCUSSION

Carbamazepine is not included among drugs causing uveitis or angle closure glaucoma. A literature search revealed one case of elevated intraocular pressure associated with carbamazepine in a patient with pseudoexfoliation glaucoma.^[8]^

**Table 1:** Diagnostic criteria for DRESS syndrome


**Bocquet et al${}^{\left[1\right]}$**	**J-SCAR**	**RegiSCAR**
All the three criteria are required	Typical DRESS: all the seven criteria are required Atypical DIHS: only the first five are required	Presence of more than three of the following criteria
1. Cutaneous drug eruption	1. Maculopapular rash developing three weeks after the treatment with the offending drug	1. Hospitalization
2. Hematologic abnormalities: Eosinophils ≥ 1.5 × 10${}^{9}$/L and the presence of atypical lymphocytes	2. Prolonged clinical symptoms after discontinuing the causative drug	2. Acute rash
3. Systemic involvement: lymphadenopathy > 2 cm, hepatitis with transaminase levels twice the normal, interstitial nephritis, pneumonitis, and carditis	3. Fever > 38${}^{\text{o}}$C	3. Reaction suspected to be drug-related
	4. Hepatic abnormalities (ALT >100 U/L) or involvement of another organ	4. Fever > 38${}^{\text{o}}$C
	5. Leukocytosis, eosinophils ≥ 1.5 × 10${}^{9}$/L, and the presence of atypical lymphocytes (> 5%)	5. Lymphadenopathy involving at least two sites
	6. Lymphadenopathy	6. Involvement of one internal organ (liver, kidney, or others)
	7. Reactivation of HHV-6	7. Hematologic abnormalities: lymphocyte count above or below normal limits, eosinophil count above laboratory limits, platelet count below laboratory limits.
	
	
ALT, Alanine transaminase; DIHS, drug-induced hypersensitivity syndrome; HHV, human herpes virus		

Aromatic anticonvulsants, such as carba- mazepine, phenytoin, and phenobarbitone, are the most common causes of DRESS; it usually occurs three weeks to three months after starting the drug therapy.^[9]^ Although many children receive anticonvulsant therapy, DRESS is rare in childhood, with fewer cases reported in children compared to adults.^[10]^ The exact pathogenesis of this syndrome is poorly understood. The implicated mechanisms include abnormalities in drug metabolism with accumulation of reactive metabolites, genetic predisposition of individuals to certain human leukocyte antigen (HLA) haplotypes, and interactions between herpes viruses and antiviral and drug-specific immune responses.^[11]^

Currently, there is no consensus on the best diagnostic criteria for DRESS syndrome. The original criteria were proposed by Bocquet et al.^[1]^ Two separate scoring systems based on the diagnostic criteria have been developed by the Japanese Research Committee on Severe Cutaneous Adverse Reaction (J-SCAR) and the European Registry of Severe Cutaneous Adverse Reaction (RegiSCAR).^[11,12]^ The RegiSCAR, which outlines seven inclusion criteria, is the most widely used [Table 1].

Our patient fulfilled three of the four main criteria for the diagnosis of DRESS syndrome (as proposed by the European RegiSCAR), namely, acute rash, fever > 38${}^{\text{o}}$C, and abnormal eosinophil and lymphocyte counts. No internal organs were involved. This can be attributed to the timely discontinuation of carbamazepine and prompt initiation of steroid therapy. Differential diagnosis in our patient includes viral infections, such as dengue, chikungunya, and hematological disorders, such as leukemia, anemia, and thrombocytopenia, all of which can present with retinal hemorrhages, serous retinal detachment, and uveitis.^[13,14,15]^

A clear temporal relationship between the administration of carbamazepine and onset of symptoms (three weeks), peripheral blood picture, an absence of findings in the clinical history and examination of the child suggestive of infection or neoplasia, and bilateral acute onset myopia with angle closure prompted us to suspect a drug-induced adverse reaction. The assessment of causality using the Naranjo Adverse Drug Reaction Probability Scale established a “probable" relationship (score of 6) with carbamazepine in the patient.^[16]^

Ocular involvement is a rare feature of DRESS syndrome and to the best of our knowledge, only five cases have been reported in literature. Uveitis (anterior, intermediate, and panuveitis) has been noted in all the cases. Reactivation of herpes viruses (human herpesvirus 6 and Epstein-Barr virus) were noted in two patients who had multi-organ involvement.^[3,4,5,6,7]^

We assume that the inflammation of the uveal tract and accumulation of exudate had resulted in uveal effusion. This led to the anterior displacement of the iris and lens diaphragm, eventually resulting in acute onset myopia, shallow anterior chamber, appositional angle closure, and elevation of intra ocular pressure in our patient. Cycloplegics promoted posterior rotation of the iris-lens diaphragm, and steroid therapy attenuated the allergic-type drug reaction, thereby stabilizing the blood-aqueous barrier and ensuring prompt recovery.

In contrast to other adverse drug reactions, dynamic changes in immune response have been reported in DRESS syndrome.^[17]^ Hence, the uveal tract with its rich network of macrophages and major histocompatibility complex (MHC) class II dendritic cells is most likely to be affected in patients with ocular involvement.

Ophthalmologists should be aware that hypersensitivity to anticonvulsants, such as carbamazepine, can present with bilateral uveitis and uveal effusion along with systemic symptoms. Given the delayed onset after the initiation of treatment with the offending drug, systemic symptoms, and potentially fatal multi-organ involvement, a high index of suspicion is necessary to prevent complications. Additionally, it is necessary to inform the patient about the adverse effects of carbamazepine before prescribing it.

##  Financial Support and Sponsorship

Nil.

##  Conflicts of Interest

There are no conflicts of interest.
